# A Scoping Review of Primary Breast Cancer Risk Reduction Strategies in East and Southeast Asia

**DOI:** 10.3390/cancers17020168

**Published:** 2025-01-07

**Authors:** Filipa Alpeza, Christine Kim Yan Loo, Qingyuan Zhuang, Mikael Hartman, Serene Si Ning Goh, Jingmei Li

**Affiliations:** 1 Genome Institute of Singapore, Agency for Science, Technology and Research (A*STAR), Singapore 138672, Singapore; filipa.alpeza@stud.ki.se (F.A.); christine.loo@u.nus.edu (C.K.Y.L.); 2 Division of Supportive and Palliative Care, National Cancer Centre Singapore, Singapore 168583, Singapore; zhuang.qingyuan@singhealth.com.sg; 3 Data Computational Science Core, National Cancer Centre Singapore, Singapore 168583, Singapore; 4 Saw Swee Hock School of Public Health, National University of Singapore and National University Health System, Singapore 117549, Singapore; mikael_hartman@nuhs.edu.sg (M.H.); serene_sn_goh@nuhs.edu.sg (S.S.N.G.); 5 Department of Surgery, National University Hospital and National University Health System, Singapore 119074, Singapore; 6 Department of Surgery, Yong Loo Lin School of Medicine, National University of Singapore and National University Health System, Singapore 117597, Singapore; 7 National Cancer Centre Singapore, SingHealth, Singapore 168583, Singapore

**Keywords:** intervention, risk reduction, cancer prevention, breast cancer, chemoprevention, surgery, Asia, scoping review, risk-based screening, risk prediction, prophylactic, tamoxifen, lose-dose tamoxifen

## Abstract

Breast cancer (BC) screening enables early detection and timely treatment of cancer. Improving the effectiveness of BC screening can be accomplished by personalizing screening schedules according to each woman’s specific risk level. However, when informing women about their risk classification, especially those at high risk, it is important to give clear recommendations on how to lower their risk. BC risk reduction comprises lifestyle modifications, preventive surgery, and chemoprevention, with the latter two being particularly applicable to high-risk individuals. Public health guidance on risk-reducing interventions is heterogeneous and context-dependent. We conducted a scoping review on BC surgical interventions and chemoprevention in East and Southeast Asia in publications between 2010 and 2024. We searched two databases and identified 23 publications relevant for inclusion. The highest number of publications came from South Korea (*n* = 9). More publications discussed surgical interventions compared to pharmacological interventions. The studies were largely observational and utilized data from medical records. Most studies defined high-risk individuals as *BRCA* carriers, many of whom previously had cancer. The field would benefit from randomized studies of BC prevention strategies focusing on Asian populations. Future research could explore women’s sentiments towards chemoprevention compared to prophylactic surgery and could extend the definition of high-risk individuals beyond *BRCA* carriers.

## Simple Summary

This study summarizes the current landscape of breast cancer risk reduction strategies in East and Southeast Asia, especially in the context of the rising interest in risk-based screening. Clinical trial results and European guidelines may not always be directly applicable to Asian populations due to differences in healthcare systems, disease prevalence, lifestyle factors, and genetic predispositions. By focusing on regional evidence, we can potentially identify gaps and areas where Asian populations may benefit from new or modified approaches. Asian evidence can inform more relevant and effective local practices.

## 1. Introduction

Breast cancer is a global public health issue, responsible for approximately 25% of all cancer diagnoses among women [1]. Despite the advancements in cancer therapies, the burden of breast cancer is expected to increase in the coming years due to the rising life expectancy worldwide [2]. Prevention and early detection have thus become pillars of cancer management, with many countries having national breast cancer screening programs. Most programs involve regular mammograms for women who have passed a specific age threshold, a practice supported by randomized controlled trials (RCTs) [3,4,5,6]. However, these programs have been criticized for failing to acknowledge the individual differences in cancer risk, as it has been well established that factors such as family history, genetic mutations, estrogen exposure, BMI, physical activity, and substance use affect one’s likelihood of developing cancer [5,7,8,9,10,11,12,13,14].

In response to those critiques, there has been a growing interest in shifting from age-based to personalized screening approaches [15,16,17]. For example, many countries have been trialing screening programs that utilize genetic, medical, and lifestyle data in risk prediction models to segment women into distinct risk categories [7,12,14,18,19,20,21]. In this way, those categorized as high risk could start attending mammograms earlier or screen more frequently than those whose risk of breast cancer is low [22,23]. Although risk-based screening might be more efficient, there are ethical implications of making women aware of their risk. The knowledge of risk classification might evoke psychological distress and a desire to act on the results, so the ethical permissibility of informing one of having a high risk of cancer without explaining how to lower it might be questioned [24,25,26,27,28].

The interventions for breast cancer risk reduction are usually split into three areas: chemoprevention, prophylactic surgery, and lifestyle modifications [29]. Chemoprevention refers to the use of pharmaceuticals to mitigate cancer occurrence; for breast cancer, selective estrogen receptor modulators (e.g., tamoxifen, raloxifene) and aromatase inhibitors (e.g., anastrozole, exemestane) have been the most promising [30,31,32,33,34]. According to the UK’s National Institute for Health and Care Excellence (NICE) eligibility criteria and assuming a 25% uptake rate, it is estimated that offering risk-reducing medication to women over 50 could prevent 11 cases of breast cancer per 1000 women [17,35]. The surgical approaches to breast cancer prevention include the prophylactic removal of entire breasts (risk-reducing mastectomy, RRM) and of ovaries and fallopian tubes (risk-reducing salpingo-oophorectomy, RRSO) [36]. RRM is reported to reduce breast cancer risk by at least 95% among women with a disease-causing BRCA mutation, while RRSO is estimated to reduce breast cancer risk by approximately 50% [37,38,39,40]. When it comes to lifestyle, typical recommendations include maintaining a healthy body mass index (BMI), limiting alcohol consumption, and engaging in regular physical activity [41,42]. However, despite their proven benefits [43], lifestyle modifications may not provide sufficient reassurance to high-risk individuals, especially those whose risks are primarily driven by genetic factors, and who might therefore prefer more targeted approaches [44].

So far, most studies on chemopreventive agents for breast cancer have been conducted in Europe and the United States, making their generalizability to Asian populations uncertain [45]. For example, recent reviews on anti-cancer drugs have highlighted that the tolerance and efficacy of some chemotherapeutic drugs might differ between populations of different ancestries [46]. Additionally, there are notable differences in the presentation of breast cancer cases in Asia compared to Western countries [47]. One of the most striking discrepancies is in the age of disease onset, given that the incidence of breast cancer in Asia seems to peak when women are in their forties and fifties, compared to the sixties and seventies in the West [6,48]. This might impact the appropriateness of surgical interventions. For example, age affects how patients view the relevance of surgical interventions. Younger women may be more concerned with physical appearance and the psychological effects of surgery, whereas older women may prioritize other quality-of-life factors after the procedure [49,50,51,52]. Furthermore, differences in cultural beliefs and system-level issues, such as infrastructure and access, might affect the acceptability and availability of preventive interventions [53,54,55,56]. For example, access rates to radiotherapy are approximately 4.7% in Cambodia, 7.9% in Myanmar, and 8.7% in Indonesia [57,58,59]. This is compounded by challenges in cancer data collection systems, poor data quality, inadequate infrastructure, and limited reporting in some countries [60,61]. As a result, these countries may prioritize immediate treatment over long-term prevention strategies because they lack the reliable data needed to guide comprehensive prevention efforts.

A scoping review of literature from East and Southeast Asia on primary breast cancer prevention helps to identify region-specific gaps in research and practice. This region has unique genetic, cultural, and healthcare factors that influence prevention strategies, yet much of the current evidence is based on Western populations. The aims of this scoping review are to (1) map the nature of East and Southeast Asian publications on chemoprevention and risk-reducing surgery for breast cancer, and (2) identify potential gaps in the literature in this context, thus providing insights relevant for stakeholders involved in breast cancer screening and prevention.

## 2. Methods

Due to the complex and multifaceted nature of the topic, we conducted a scoping review of the literature on pharmacological and surgical interventions for reducing the risk of breast cancer in East and Southeast Asia. This approach allows us to capture the variety of study designs and disciplinary perspectives within the field, as well as to identify key concepts and gaps [62]. The review was guided by the Arksey and O’Malley framework for scoping studies, which comprises five stages: (1) identifying the research question, (2) identifying the relevant studies, (3) study selection, (4) charting the data, and (5) collating, summarizing, and reporting the results [63]. We reported this study following the Preferred Reporting Items for Systematic Reviews and Meta-Analyses (PRISMA) extension for scoping reviews checklist [64].

### 2.1. Search Strategy

The search was performed in two online databases, PubMed and Web of Science Core Collection, on 11 July 2024. PubMed provides a comprehensive coverage of medical literature, including clinical trials and observational studies. PubMed also offers advanced filtering options, making it ideal for navigating its structured database for precise queries [65]. Web of Science provides broader multidisciplinary research coverage, including both clinical and policy-based breast cancer prevention strategies [66]. We restricted the search to articles published in English since 2010 to increase the relevance of our findings [67]. The full search strategy for both databases is available in Supplementary Tables S1 and S2.

### 2.2. Eligibility and Study Selection

To be included in this review, studies needed to meet the following inclusion criteria: (1) be based in an East or Southeast Asian country, (2) discuss chemoprevention and/or risk-reducing surgery for breast cancer, and (3) report strategies that show potential for lowering breast cancer risk. We excluded studies that recruited only women who already had cancer, studies that paid limited or no attention to breast cancer, and those for which the full text was unavailable. The rationale for not including articles that studied only breast cancer patients was to make our findings more relevant for risk management in the context of breast cancer screening programs, which target healthy individuals. There was no restriction on the type of study design eligible for inclusion.

We exported all search results into EndNote (v 21.2 bld 19537) to remove duplicates. The unique records were then transferred to Rayyan, a web-based systematic review software, for article screening [68]. In the first round of screening, all titles and abstracts were blindly reviewed by two authors (F.A. and C.K.Y.L.). Any conflicts were resolved in a team discussion before progressing to the second round of screening. In this round, the two authors independently assessed the full texts of the remaining studies against the inclusion criteria. The authors subsequently compared their decisions and agreed on which studies aligned with the scope of this review, and noted the specific reasons for article exclusions. The screening processes were graphically summarized using a PRISMA flow diagram [69].

### 2.3. Data Extraction and Analysis

From the included articles, we extracted information on the title, authors, year of publication, location, aims, methods, number and characteristics of participants, risk-reduction method, and main findings. The data were charted into an extraction table in MS Excel. Having collated all the relevant information from the studies, we summarized the key findings using two approaches. First, we graphically presented the overarching features of the included studies, focusing on their geographical distribution, publication date, and type of intervention discussed. Second, we thematically grouped the studies based on the risk-reduction strategies they covered (risk-reducing surgery, chemoprevention, or both surgery and chemoprevention). We then produced a narrative account of the key messages from each group. Organizing the data this way enabled us to gauge the dominant sources of knowledge in the field, perform intra- and inter-group comparisons, and observe what evidence might be missing.

## 3. Results

### 3.1. Scope of Studies

**Figure 1:**
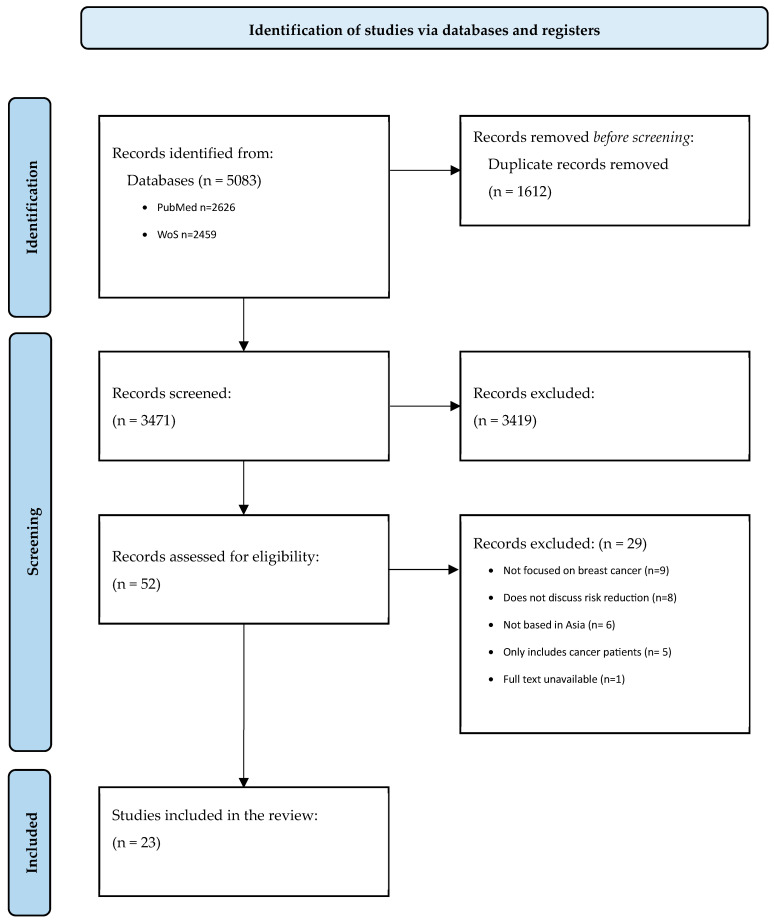
Flowchart of included studies.

**Figure 2:**
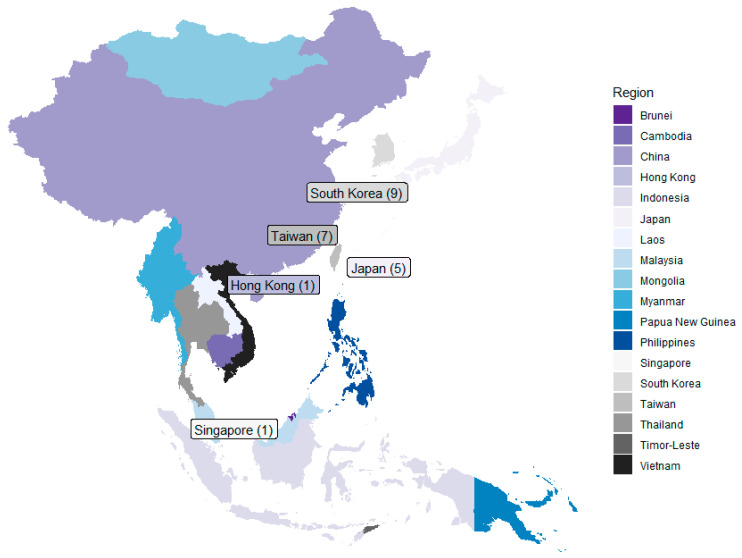
Geographical distribution of articles included. The number of articles is denoted within parentheses.

Our search resulted in 5083 records, of which 3471 remained after de-duplication (Figure 1). Following the title and abstract screening, we assessed the full text of 52 studies for eligibility. During the first round of screening, we noted that most studies on risk reduction of breast cancer in Asia focused on dietary patterns—particularly, consumption of soy. In the second round of screening, the most common reason for the exclusion of records was a lack of focus on breast cancer (*n* = 9). At the end of the screening process, 23 publications were included in this review (Figure 2 and Table 1).

### 3.2. Geographical Distribution

The majority of publications were from South Korea (*n* = 9, IDs 4, 5, 24, 27, 30, 31, 38, 61, 63), Taiwan (*n* = 7, IDs 39, 53, 95, 96, 97, 111, 112), and Japan (*n* = 5, study IDs 28, 49, 59, 62, 110). The sample sizes reported in the included studies ranged from 1 (ID 49) to >400,000 (*n* = 3, IDs 95, 96, 97). All studies from Taiwan focused on women with type II diabetes and studied potential chemopreventive agents.

### 3.3. Study Designs and Methodologies

Most studies were retrospective cohort or case–control studies that analyzed information from existing databases (*n* = 16, IDs 12, 24, 27, 30, 31, 38, 39, 53, 59, 61, 62, 95, 96, 97, 111, 112). The remaining study types were cross-sectional studies (*n* = 4, IDs 4, 5, 37,63), a case report (ID 49), an economic evaluation (ID 110), and clinical practice guidelines (ID 28).

### 3.4. High-Risk Groups Targeted

Ten studies focused on cancer risk reduction among carriers of BRCA gene mutations (IDs 4, 24, 27, 30, 37, 38, 49, 59, 62, 110), and it was common for studies to compare risk-reducing behaviors of affected and unaffected BRCA carriers. Two studies included only individuals without a personal history of breast cancer (IDs 49, 63).

### 3.5. Prophylactic Surgery

More studies explored risk-reducing surgery as a method of prevention of breast cancer (*n* = 11, IDs 4, 5, 12, 24, 27, 30, 49, 59, 62, 63, 110) compared to chemoprevention (*n* = 9, IDs 31, 39, 53, 61, 95, 96, 97, 111, 112). Three publications discussed both approaches to risk reduction (IDs 28, 37, 38).

Studies have shown that women with a personal history of breast cancer have significantly different approaches to cancer risk reduction compared to those without (*n* = 5, IDs 12, 27, 30, 38, 62). In a study investigating trends in risk-reducing surgery in South Korea (ID 27), RRM was performed in 9.9% and RRSO was performed in 34.6% of affected BRCA carriers. In comparison, 1.2% and 11.9% of BRCA carriers without a history of breast cancer underwent RRM and RRSO, respectively. RRM seemed more common among younger women (IDs 37, 62, 63), while RRSO became more acceptable with increasing age (IDs 4, 30, 38). For example, study ID 4 involved 52 BRCA mutation carriers who were at least 35 years old and found that 57% opted for RRSO. Those who underwent RRSO were significantly older than those who did not. Another study (ID 12) assessed adherence to risk management recommendations among patients who received a genetic test result showing the presence of cancer-associated mutation. Adherence was defined as undergoing RRM or annual breast imaging. The study found 74% of patients to be fully adherent, but the adherence decreased with age. Similarly, ID 63 found that the intention to undergo RRM decreased with age among cancer-free Korean women carrying BRCA mutations (odds ratio [OR] 0.39; 95% CI, 0.20 to 0.74 and 0.30; 95% CI, 0.14 to 0.61, for 30–34-year-olds and 35–39-year-olds, respectively, compared to 20–24-year-olds). Study ID 5 surveyed a cohort comprising the public, cancer patients, researchers, and clinicians using hypothetical BRCA testing scenarios and reported that 36% expressed intent to undergo RRSO, while 27% expressed intent to undergo RRM. For both types of prophylactic surgery, the most common barrier to uptake was not having a personal history of breast cancer. In addition to one’s medical history, other factors that might influence the decision to undergo surgery include the perceived risk of cancer and education level (IDs 5, 24).

There have been several accounts of the rise in genetic testing and risk-reducing surgery for breast cancer, as well as changes in insurance coverage for these procedures (IDs 27, 38, 59). Risk-reducing surgeries were found to be cost-saving and more effective than surveillance among high-risk individuals (ID 110). In general, women seemed to be more open to risk-reducing surgery than chemoprevention when it came to managing their breast cancer risk (IDs 37, 38). For example, a Korean study (ID 38) involving 179 female BRCA carriers found that 10.1% received chemoprevention (olaparib, tamoxifen, or oral contraceptives), while 44.7% underwent risk-reducing surgery. However, it should be noted that tamoxifen may have limited efficacy in preventing ER-negative and triple-negative tumors, which are more common in BRCA-related cancers [91].

### 3.6. Chemopreventive Agents

Research on chemopreventive agents for breast cancer in Asian populations appears limited, with more observational data than randomized controlled studies. Observational evidence suggests potential risk-reducing properties for several agents, including tamoxifen (ID 31), aspirin (ID 111, 112), ACE inhibitors combined with aspirin or NSAIDs (ID 39), analgesics (ID 61), sitagliptin (ID 96), and metformin alone (ID 95) or with rosiglitazone (ID 97). The potential of statins to lower the risk of cancer was limited and the data were inconclusive (ID 53).

Out of the nine papers that studied chemoprevention, seven were based in Taiwan (IDs 39, 53, 95, 96, 97, 111, 112), and five of those included only patients with type II diabetes (IDs 95, 96, 97, 111, 112) listed in the National Health Insurance database. There was an apparent dose–response relationship between metformin use and breast cancer risk (IDs 95, 97). It is hypothesized that metformin may reduce breast cancer risk by lowering insulin levels or activating AMPK to inhibit cancer cell growth [92]. Although supported by some studies, this relationship is currently not well established, and it possibly differs based on tumor hormone receptor status and the patient’s metabolic profile [92,93]. Diabetic patients using aspirin appeared to have a decreased risk of hormone receptor-positive breast cancer (IDs 111, 112), which aligns with findings from a meta-analysis of observational studies on prophylactic aspirin intake and breast cancer risk [94]. Study ID 112 noted that a cumulative dose of more than 8600 mg of aspirin for a mean period of 8.5 years led to a reduction in the risk of HR+ cancer. Similar effects on HR– cancer were observed only if the cumulative dose of aspirin exceeded 88,900 mg.

Based on the data from 25,992 breast cancer patients and age-matched controls in Taiwan (ID 53), exposure to lovastatin, a lipophilic statin, within three years before the date of diagnosis was associated with lower odds of breast cancer (adjusted OR 0.596, 95% CI 0.497–0.714). However, no significant difference in odds ratios for breast cancer was observed for users of non-lipophilic statins such as simvastatin, pravastatin, fluvastatin, and atorvastatin.

ID 61 found an association between regular analgesic use and a lower risk of breast cancer compared with never using analgesics (hazard ratio [HR] 0.748, 95% CI: 0.614–0.912) in a sample of 6735 Korean cancer patients and cancer-free controls of working age. Chemoprevention with tamoxifen was explored in a study (ID 31) that estimated the risks and benefits of using the drug based on information from Korean medical databases. The study found that, at an average risk of breast cancer, the risk–benefit index of tamoxifen was positive only for women younger than 40.

ACE inhibitors may aid in cancer prevention by lowering angiotensin II levels, which are associated with tumor growth and angiogenesis [95]. A nation-wide Taiwanese study (ID 39) found that ACE inhibitors alone were not significantly associated with breast cancer risk. However, when combined with NSAIDs, women who were exposed to the highest cumulative doses of both drugs (more than 365 cumulative defined daily doses within their respective study period) had a lower risk of breast cancer (adjusted OR 0.60, 95% CI 0.52–0.70). Similarly, a significant reduction in breast cancer risk was observed among women exposed to the highest cumulative doses of ACE inhibitors combined with aspirin.

A commonly reported limitation of the included studies was a small sample size (*n* = 7, IDs 4, 12, 24, 30, 37, 49, 59). The studies that analyzed health records highlighted issues such as imprecision of record data (e.g., uncertainty around adherence to prescriptions) and a lack of information on factors that could act as confounders for the development of breast cancer, such as BMI, age of menarche, and perceived risk.

## 4. Discussion

The review identified 5083 studies related to breast cancer risk reduction. However, the final analysis included only 23 publications on surgical and pharmacological interventions for reducing breast cancer risk in East and Southeast Asia. Most studies were observational and retrospective, defined high-risk individuals as BRCA mutation carriers, and were more often on surgical interventions compared to chemopreventive ones. During the screening process, we excluded a substantial number of Asian publications on breast cancer prevention through dietary modification. The apparent scholarly interest in this area might be present due to the high soy consumption in Asia, which has shown protective effects against breast cancer [96]. The effect of diet on breast cancer risk has been summarized in other studies and was beyond the scope of this review [97,98].

### 4.1. Prevalence of Surgical Risk Reduction over Chemoprevention

Our search captured more literature on surgical than pharmaceutical breast cancer risk reduction in East and Southeast Asia. This may be due to events occurring within the publication timeframe applied to this review (years 2010–2024) that increased awareness of primary cancer prevention with surgery. For example, some papers (ID 38, 63) mentioned Angelina Jolie’s prophylactic mastectomy in 2013 and her influence on individuals with elevated risk, sometimes referred to as “the Angelina Jolie effect” [99,100,101]. Such celebrity endorsement might have contributed to prophylactic surgery becoming more well known, subsequently increasing the demand [102,103]. Insurance coverage for BRCA testing and preventive surgeries might have also contributed to increased patient and research interests in surgery. For example, study ID 59 highlighted that the uptake rate of RRSO in Japan increased up to threefold after the national insurance system covered the RRSO and RRM procedures for breast cancer patients with BRCA pathological variants in April 2020. Study ID 38 showed a similar pattern in Korea after the National Health Insurance System (NHIS) subsidized RRSO for BRCA pathogenic carriers in 2013. They also noted that insurance reimbursement of RRM for breast cancer patients with a BRCA mutation was introduced in 2017, which could incentivize uptake. The increased uptake of risk-reducing surgery has also been observed outside the Asian context, and the cost-effectiveness of such surgeries for women with an increased risk of breast cancer has been reported previously [99,104,105].

Chemopreventive clinical trials appear to be more common in Europe than in Asia, indicating a divergence in research priorities and drug development across these regions. Raloxifene, a selective estrogen receptor modulator (SERM), has been widely studied in Europe for both breast cancer prevention and osteoporosis treatment. Since 1999, trials in European countries have examined raloxifene’s impact on breast cancer cell markers like Ki67 and apoptosis [106]. In contrast, clinical trials investigating raloxifene in Asia remain sparse. A nationwide study in Korea found limited evidence supporting raloxifene’s efficacy in reducing breast cancer risk and emphasized the need for more research in Asian populations [107].

Beyond raloxifene, Europe has a broader spectrum of breast cancer chemoprevention trials, including IBIS-I (tamoxifen), IBIS-II (anastrozole), MAP.3 (exemestane), and LIBER (letrozole for BRCA mutation carriers) [108,109,110,111]. In contrast, the adoption of chemoprevention is less common in Asia, where there is greater reluctance to use preventive medications [112]. While there are some efforts, such as a tamoxifen trial in Japan and China (NCT03423199), many other drugs remain underexplored. More clinical trials and RCTs are urgently needed to strengthen chemopreventive options for high-risk populations in East and Southeast Asia.

### 4.2. Factors Influencing Uptake of Risk-Reducing Surgery Versus Chemoprevention

A common finding within the surgery-centered studies was that increased age was positively associated with RRSO uptake and inversely associated with RRM uptake among BRCA carriers, which is consistent with existing literature [113]. This might be partially explained by the differences in the age of onset of breast and ovarian cancer, respectively, with ovarian cancer usually being diagnosed in older patient groups [114]. Although countries covered in this review primarily base their guidance on RRSO and RRM on one’s genetic risk, there are some age-related considerations. Surgery is usually recommended for carriers of BRCA pathogenic variants who are in their late 30s and mid-40s and have completed childbearing [78]. Nonetheless, RRSO may be delayed until a woman turns 40 if she is known to carry the BRCA2 mutation and not the BRCA1 mutation, as is the case in Japan (ID 59). The included studies also confirmed that sentiments towards risk-reducing surgery differed between affected and unaffected BRCA carriers (IDs 12, 27, 30, 38, 62) and that they might be influenced by factors such as family history of cancer, cancer-related worry, and education level [115,116]. Studies that assessed both surgical prevention and chemoprevention found that surgeries and surveillance seemed to be better accepted than medication such as tamoxifen (ID 37, 38). Although randomized trials have shown that tamoxifen reduces breast cancer risk among healthy high-risk women, hesitation towards its use has been reported, partially due to its association with serious adverse events such as endometrial cancer [117,118,119,120,121,122,123,124,125]. In addition, endocrine-targeted agents are observed to reduce hormone-positive cancers but not breast cancer deaths, prompting a reassessment of current risk reduction strategies [126].

A lack of awareness might also be contributing to the suboptimal uptake of tamoxifen [127]. A UK-based multi-center survey of healthy women considering breast cancer primary prevention found that only one out of six women understood the potential benefits and harms of tamoxifen [128]. The only study in our review that focused specifically on chemoprevention with tamoxifen attempted to calculate the risk–benefit of using the drug in Korea (ID 31). The authors concluded that tamoxifen is beneficial for Korean women younger than 40, while the standard clinical guidance is to prescribe tamoxifen to women older than 35 [76,129,130]. However, the study was limited by having to rely on efficacy data of tamoxifen in the US population due to the lack of clinical trials in Korea. In addition, while tamoxifen does not appear to directly impair ovarian function, its prolonged use can delay pregnancy plans and negatively impact fertility, especially for older reproductive-aged women. Addressing fertility concerns may improve adherence to this important breast cancer treatment. The uptake and implementation of risk reduction strategies for breast cancer in East and Southeast Asia might also be shaped by sociocultural factors. Perceptions of cancer in this region are influenced by religious heterogeneity and underlying values of conservativism and collectivism [131]. For instance, a study from Malaysia found that women’s strong trust in alternative medicine and traditional healers might lead to reservations towards Western medicine, and that cultural norms of appreciating bodily integrity might discourage them from invasive procedures such as surgery [55]. Some of the additional barriers to breast-related care include fear of pain and social judgment, feeling embarrassed, beliefs that diseases such as breast cancer are predetermined and inevitable, and a lack of support from friends and family [6].

### 4.3. Differences in the Definition of High Risk

Coupled with the relative lack of chemoprevention efficacy data in East and Southeast Asia compared to in America and Europe, studies focusing on high-risk individuals in these regions tend to have a narrower definition of risk. Many Asian studies focus on BRCA carriers as high-risk individuals, when in fact, the risk of breast cancer is contributed to by many other attributes [132,133]. In contrast, European studies also look at other factors, such as the diagnoses of atypical ductal hyperplasia (ADH) and atypical lobular hyperplasia (ALH) [134,135]. Some studies also include healthy individuals identified to be at high risk from genetic and non-genetic risk predictors, which Asian studies often do not focus on [136,137].

### 4.4. Gaps and Opportunities for Research

Based on the review of the identified studies, we noted several potential gaps in the literature. Geographically, there was limited representation outside East Asia—specifically, beyond South Korea, Taiwan, and Japan. Regarding study design, experimental studies are absent, particularly RCTs on chemopreventive agents for breast cancer in Asian populations. The studies are mainly observational and retrospective (16 out of 23), limiting the ability to infer causality. Most studies focused on BRCA gene mutations. There are other definitions of high risk, such as healthy individuals classified as high risk based on non-genetic risk factors or patients diagnosed with DCIS and atypical lesions, who may also benefit from risk-reduction interventions. It is also important to acknowledge that BRCA-negative, high-risk women might have different sentiments toward the pharmaceutical and surgical prevention strategies discussed here. Regarding prophylactic interventions, while some agents like tamoxifen, aspirin, and metformin were explored, there is a lack of studies investigating newer or less commonly used chemopreventive agents, such as raloxifene, aromatase inhibitors, selective progesterone receptor modulators (SPRMs), and other nonsteroidal anti-inflammatory drugs (NSAIDs) [138,139,140,141,142,143]. There is also limited research on how different breast cancer prevention strategies impact patient quality of life, satisfaction, and overall well-being. For example, effects on body image and psyche are often overlooked and not discussed [144]. In addition, it is important to note the uncertainty surrounding the optimal duration of treatment. The long-term administration of chemopreventive agents such as anti-inflammatories, statins, or tamoxifen, particularly in high-risk, young patients, poses challenges due to potential side effects and cumulative harm. Moreover, the lack of clarity on what happens when treatment is discontinued, especially when breast cancer risk continues to increase over time, highlights the need for further investigation into the appropriate length of treatment and follow-up for these interventions.

### 4.5. Strengths and Limitations

Asian populations are often underrepresented in global breast cancer research. To our knowledge, this scoping review of a broad spectrum of relevant studies is the first to map the literature on surgical and pharmaceutical prevention of breast cancer in East and Southeast Asia. The review also provides an overview of various chemopreventive agents studied, or lack thereof, in Asian populations. However, the review has some limitations. Despite searching two large databases and following a standardized methodological framework, we might have missed relevant studies published elsewhere or used terminology that deviated from our search. It is also possible that some studies from East and Southeast Asia are not published in English-language journals. Several studies included in the review had small sample sizes, which can lead to reduced statistical power and may limit the generalizability of the findings. Small studies are also more prone to bias and may not adequately capture the variability in outcomes. Additionally, all included studies were observational, which should be kept in mind when interpreting the results. Worthy of note is that this study design is insufficient to prove causality between an intervention and breast cancer risk reduction.

## 5. Conclusions

By focusing on East and Southeast Asia, the review helps identify gaps in the research and highlights the need for randomized controlled l studies tailored to Asian populations. While such research is important for advancing long-term prevention efforts, from a primary care perspective, recommending more frequent or earlier screening for high-risk individuals is a practical approach to breast cancer risk management. Early detection, particularly when cancers are identified at an early stage, can improve treatment outcomes and may reduce the need for more aggressive therapies, such as chemotherapy or radiotherapy. Lifestyle modifications, including weight management and dietary adjustments, are also appropriate preventive measures. However, more invasive interventions, such as prophylactic chemoprevention or surgery, should be considered primarily for individuals at very high risk. There is also a need for better data quality, comprehensive reporting standards, and inclusion of underrepresented populations across the region. To advance these efforts, we recommend prioritizing well-designed randomized controlled studies that actively involve patient participants and incorporate input from healthcare professionals. Enhancing data collection methods and fostering cross-disciplinary collaborations will be essential to developing effective, evidence-based prevention strategies tailored to diverse populations.

## Figures and Tables

**Table 1:** Summary of included studies. Articles are ordered by author names. ID denotes the corresponding position of the article during the full-text screening process.

ID	Reference	Title	Year	Location	Aim	Type of Study	Participants	Risk Reduction Method	Key Findings
4	[70]	Effect of risk-reducing salpingo-oophorectomy on the quality of life in Korean BRCA mutation carriers	2021	Korea	This study aimed to compare the QOL, psychosocial status, sexual function, and menopausal symptoms between the risk-reducing salpingo-oophorectomy (RRSO) and non-RRSO groups comprising Korean women with BRCA mutation and to evaluate the effect of timing of RRSO (before and after menopause) on those aspects.	Cross-sectional	52 women aged≥ 35 years, 47 of whom were affected and 5 of whom were unaffected carriers of BRCA mutation	Risk-reducing salpingo-oophorectomy	Opting for RRSO was associated with poorer physical quality of life only but did not negatively affect mental QOL, psychosocial status, sexual function, or menopausal symptoms. When comparing groups based on menopausal status, the only significant difference was that mental score component of QoL was higher in the premenopausal than in the postmenopausal group.
5	[71]	Differences in Willingness to Undergo BRCA1/2 Testing and Risk Reducing Surgery among the General Public, Cancer Patients, and Healthcare Professionals: A Large Population-Based Survey	2022	Korea	This study aimed to compare the intent to undergo BRCA1/2 testing, risk-reducing salpingo-oophorectomy (RRSO), and risk-reducing mastectomy (RRM) among the general public, cancer patients, and healthcare professionals in Korea.	Cross-sectional	3444 individuals; 1496 from the general public, 1500 cancer patients, 108 clinicians, and 340 researchers	Risk-reducing salpingo-oophorectomy and risk-reducing mastectomy	The main factor contributing to RRSO intent was “being at high risk of ovarian cancer” (72%). Among the barriers reported by those not intending to undergo RRSO, “the fact that I do not have breast cancer yet” (60%) was the most common. The most common reason behind the intent to undergo RRM was “test results indicated being at high risk of developing breast cancer” (67%). “The fact that I do not have breast cancer yet” (66%) was the most frequently reported reason among individuals not willing to undergo RRM.
12	[72]	Risk management adherence following genetic testing for hereditary cancer syndromes: a Singaporean experience	2018	Singapore	This study aims to examine adherence behavior to risk management guidelines among mutation carriers who have attended the Cancer Genetic Service (CGS) in Singapore.	Retrospective cohort study	52 subjects with a confirmed presence of germline mutations in a gene associated with a hereditary cancer syndrome, 41 of whom were affected carriers	Risk-reducing mastectomy	The overall adherence rate was 96.2%, including 37 (74.0%) fully adherent and 13 (26.0%) partially adherent subjects. Adherence was similar across the age groups; however, it decreased with increasing age. Malay subjects had lower adherence compared with Chinese and other ethnic groups. Those who were the first in their family to undergo testing (proband testing) demonstrated lower adherence than those who underwent testing for a known familial mutation.
24	[73]	Trends in contralateral prophylactic mastectomy rate according to clinicopathologic and socioeconomic status	2019	Korea	This study aimed to examine trends in CPM (contralateral prophylactic mastectomy) rates according to clinicopathologic and socioeconomic status at a single institution in the Republic of Korea.	Retrospective cohort study	128 participants diagnosed with BRCA mutations	Risk-reducing mastectomy	Women in the CPM group had significantly higher education levels. A total of 75% of participants who underwent CPM also underwent RRSO (compared to 12.5% in the non-CPM group).
27	[58]	Trends in Risk-Reducing Mastectomy and Risk-Reducing Salpingo-Oophorectomy in Korean Carriers of the BRCA1/2 Mutation	2020	Korea	The aim of this study was to investigate trends in risk-reducing surgery for the prevention of breast and ovarian cancer in Korean carriers of the BRCA1/2 mutation until August 2018.	Retrospective cohort study	1238 affected and 514 unaffected BRCA mutation carriers	Risk-reducing salpingo-oophorectomy and risk-reducing mastectomy	Contralateral RRM among affected carriers increased approximately 5.8-fold, from 5 cases in 2013 to 29 cases in 2017. RRBSO among affected carriers also increased approximately 3.6-fold, from 22 cases in 2013 to 79 cases in 2017. Among unaffected carriers, bilateral RRM increased by 1 case in 2017, and RRBSO increased 8-fold from 2 cases in 2013 to 16 cases in 2017.
28	[74]	The Japanese Breast Cancer Society clinical practice guidelines for epidemiology and prevention of breast cancer, 2022 edition	2024	Japan	To provide a summary of the 2022 edition of the JBCS Clinical Practice Guidelines for Epidemiology and Prevention of Breast Cancer	Clinical practice guidelines	Not applicable	Chemoprevention, risk-reducing mastectomy, risk-reducing salpingo-oophorectomy	Prophylactic administration of selective estrogen receptor modulators (tamoxifen, raloxifen) or aromatase inhibitors (exemestane, anastrozole) to women at high risk of breast cancer has been found to prevent onset of breast cancer. However, a breast cancer risk model for Japanese women has not been established, so it is not possible to conclude whether administration of drugs to prevent development of breast cancer is or is not useful. Bilateral risk-reducing mastectomy (BRRM) is weakly recommended for breast cancer-naive women with BRCA pathologic variants. There is a weak recommendation for contralateral risk-reducing mastectomy (CRRM) in patients with a diagnosis of breast cancer with a BRCA pathologic variant. RRSO is strongly recommended for women with BRCA mutations. In Japan, the medical reimbursement revision in April 2020 allows for risk-reducing mastectomy (RRM) and risk-reducing surgery for oophorectomy (RRSO) covered by insurance.
30	[75]	Factors affecting the decision to undergo risk-reducing salpingo-oophorectomy among women with BRCA gene mutation	2013	Korea	This study evaluated the clinical and demographic factors that may affect the decision to undergo RRSO among South Korean women with BRCA gene mutations.	Retrospective cohort study	71 BRCA mutation carriers older than 30, 51 of whom were affected carriers	Risk-reducing salpingo-oophorectomy	The RRSO rate of carriers in their fifth decade was significantly higher than that of the other age groups (*p* = 0.007). While 20 of 51 (39.2%) personally affected carriers had undergone RRSO, only 1 of the 20 (5.0%) personally unaffected carriers had undergone RRSO (*p* = 0.004). This revealed that whereas a personal history of breast cancer was a significant factor in the decision to undergo RRSO among the carriers, BMI and family history of ovarian cancer or breast cancer were not, and neither were alcohol intake, cigarette smoking, occupation, or economic status.
31	[76]	Estimating the Risks and Benefits of Tamoxifen for Prophylactic Breast Cancer Chemoprevention in Korea	2012	Korea	This study estimated the risks and benefits of tamoxifen using a Korean database in order to evaluate the feasibility of using tamoxifen for chemoprevention in Korean women.	Retrospective cohort study	Women from Korean National Insurance Database	Chemoprevention with tamoxifen	For the specific 5-year risk of breast cancer, the net benefit of tamoxifen was reduced based on increases in age because of the high risk of stroke in older women. Therefore, older Korean women had more risk than benefit from tamoxifen chemoprevention. Only women younger than age 40 had a positive risk–benefit index for the average 5-year risk of breast cancer.
37	[77]	Choice of Management of Southern Chinese BRCA Mutation Carriers	2010	Hong Kong	This study was designed to be the first to report the uptake of preventative and surveillance measures of Chinese women and men who were found to be carriers of the BRCA1 and BRCA2 mutations.	Cross-sectional	31 affected carriers of the BRCA mutations and 83 of their family members	Risk-reducing surgeries and chemopreventative agents (tamoxifane, raloxifane, birth control)	Women who decided on prophylactic salpingo-oophorectomy were comparatively older than those who decided on prophylactic mastectomy. Interestingly, no female mutation carriers, excluding those who were already taking tamoxifen, agreed to have any chemoprevention.
38	[78]	Different Patterns of Risk Reducing Decisions in Affected or Unaffected BRCA Pathogenic Variant Carriers	2019	Korea	This study was designed to identify risk reduction management patterns in Korean carriers of BRCA1 and BRCA2 pathogenic variants.	Retrospective cohort study	220 BRCA mutation carriers, 100 of whom were affected	Risk-reducing surgery and chemoprevention	Of the 179 female carriers, 98 (54.7%) underwent RR management, including 18 (10.1%) who received chemoprevention and 80 (44.7%) who underwent risk-reducing surgery (RRS), including RRSO in 76 patients, RRM in 1 patient, and both in 3 patients. Of the 79 unaffected female carriers, 39 (49.4%) received RR management, including 23 (29.1%) who underwent RRSO and 16 (20.3%) who received chemoprevention (*p* = 0.495). In affected female carriers with BC, older age was significantly associated with RR management (*p* = 0.011). However, in unaffected carriers, age, type of BRCA pathogenic variant, and family history were not significantly associated with RR management.
39	[79]	Angiotensin-converting enzyme inhibitors enhance the effect of cyclooxygenase inhibitors on breast cancer: a nationwide case-control study	2012	Taiwan	This study aimed to investigate the effects of NSAIDs, aspirin, and ACE inhibitors, alone or in combination, on the risk of developing breast cancer.	Case–control	A total of 16,847 breast cancer patients and 50,541 age-matched, cancer-free controls from the Taiwanese NHI database	Chemoprevention with NSAIDs, aspirin, and ACE inhibitors—alone or in combination	There were significantly lower rates of breast cancer among women who took aspirin and high doses of ACE inhibitors compared with women taking aspirin and lower doses of ACE inhibitors. Women taking NSAIDs and higher doses of ACE inhibitors also had significantly lower rates of breast cancer compared with women taking NSAIDs with lower doses of ACE inhibitors. Increasing doses of ACE inhibitors alone had no effect on breast cancer prevention.
40	[80]	Experience with Bilateral Risk-Reducing Mastectomy for an Unaffected BRCA Mutation Carrier	2016	Japan	This paper reports a case of a 38-year-old Japanese woman diagnosed with BRCA2 mutation that underwent prophylactic bilateral SSM.	Case report	A Japanese woman who was an unaffected carrier of BRCA2 mutation	Risk-reducing mastectomy	Not applicable; descriptive account of the case
53	[81]	Lovastatin lowers the risk of breast cancer: a population-based study using logistic regression with a random effects model	2016	Taiwan	This study aimed to statistically evaluate the risk of BC for statin users and non-users in Taiwan, as well as for each statin.	Case–control	A total of 4332 breast cancer patients and 21,660 cancer-free controls from the Taiwanese NHI database	Chemoprevention with statins (atorvastatin, fluvastatin, lovastatin, pravastatin, simvastatin)	The adjusted odds ratio for BC among lovastatin users was 0.596 (95% CI 0.497–0.714), lower than that for subjects who did not use lovastatin. Simvastatin, pravastatin, fluvastatin, and atorvastatin did not exhibit a statistically significant protective effect against BC, although atorvastatin showed a protective tendency against BC that did not reach statistical significance (adj OR 0.887; 95% CI 0.776–1.013; *p* < 0.077).
59	[57]	Impact of the coverage of risk-reducing salpingo-oophorectomy by the national insurance system for women with BRCA pathogenic variants in Japan	2023	Japan	To determine the impact of the coverage of risk-reducing salpingo-oophorectomy (RRSO) by the national insurance system by comparing cases before and after the coverage date	Retrospective cohort study	134 women underwent with confirmed BRCA mutations	Risk-reducing salpingo-oophorectomy	Before insurance coverage, 45 women underwent RRSO in 36 months, whereas 89 women underwent RRSO in 24 months after coverage. The median age, BRCA status, personal BC history, and family history of OC to within the third degree between the groups were not statistically different. There were significantly more parous women after coverage.
61	[82]	Association between WHO First-Step Analgesic Use and Risk of Breast Cancer in Women of Working Age	2023	Korea	To analyze the association between economic activity, regular analgesic use, and breast cancer development in women of specific age groups to provide a basis for improving cancer prevention practices	Retrospective cohort study	Women of working age from The Korean National Health Insurance Service—National Sample Cohort database, 1347 of whom were cancer patients and 5388 of whom were not	Chemoprevention with analgesics	The economically active group had a higher risk of breast cancer than the inactive group (*p* < 0.001), possibly due to higher levels of stress and lower engagement in health protective behaviours. The risk of breast cancer was higher in the high-income group than in the low-income group (*p* < 0.001). The risk of breast cancer was lower in participants with regular analgesic use compared to those with no analgesic use (HR = 0.748, 95% CI: 0.614–0.912, *p* < 0.05). Regular analgesic use may be beneficial for inhibiting cancer development in the young age group (24~39 years).
62	[83]	Risk-reducing mastectomy for women with hereditary breast and ovarian cancer (HBOC): analytical results of data from the Japanese Organization of HBOC	2022	Japan	To confirm the rate of risk-reducing mastectomy in women with BRCA pathogenic variants before the approval of National Medical Insurance coverage	Retrospective cohort study	687 women with confirmed BRCA mutations	Risk-reducing mastectomy	The rates of RRM were statistically significantly higher in women with a personal (positive 15.4% vs. negative 5.2%, *p* = 0.001) or family history (positive 16.6% vs. negative 10.5%, *p* = 0.042) of breast cancer than those without them, in mothers compared with those without children (mothers 15.1% vs. women without children 7.9%, *p* = 0.023), in women who were receiving surveillance with MRI than women who did not (surveillance +23.9% vs. surveillance −9.7%, *p* < 0.001), and in women who received RRSO compared with women who did not (RRSO +36.7% vs. RRSO −7.3%, *p* < 0.001).
63	[84]	Attitudes toward Risk-Reducing Mastectomy and Risk-Reducing Salpingo-oophorectomy among Young, Unmarried, Healthy Women in Korea	2022	Korea	To investigate the attitudes toward risk-reducing mastectomy (RRM) and risk-reducing salpingo-oophorectomy (RRSO) as cancer prevention options for BRCA1/2 carriers in healthy, young, unmarried Korean women	Cross-sectional	600 cancer-free unmarried women aged 20–39 from different parts of South Korea	Risk-reducing mastectomy and risk-reducing salpingo-oophorectomy	Among 600 women aged 20–39 years, 54.7% and 57.7% had the intention to undergo RRM and RRSO, respectively, on the assumption that they were BRCA1/2 carriers. As age increased, the proportion of women with the intention to undergo RRM or RRSO decreased from >65% in the 20–24-year-old age group to around 40% in the 35–39-year-old age group. Those who wanted to undergo screening for BRCA1/2 mutations had a significantly high intention to undergo risk-reducing surgeries (*p* < 0.05).
95	[85]	Metformin may reduce breast cancer risk in Taiwanese women with type 2 diabetes	2014	Taiwan	To evaluate whether metformin use in the Taiwanese women with T2DM would affect the risk of breast cancer	Retrospective cohort study	476,282 patients with a diagnosis of newly onset type II diabetes mellitus	Chemoprevention with metformin	For the overall hazard ratios comparing ever users versus never users, there was a significantly lower risk of breast cancer associated with metformin use. In the dose–response analyses adjusted for age, all categories of exposure to metformin were associated with a significantly reduced risk. In the fully adjusted models, although a significantly reduced risk was observed with increasing cumulative duration and cumulative dose, the first tertiles of the dose–response parameters showed a significantly higher risk associated with metformin use.
96	[86]	Sitagliptin May Reduce Breast Cancer Risk in Women With Type 2 Diabetes	2017	Taiwan	To evaluate whether sitagliptin use in Taiwanese female patients with T2DM would affect the risk of breast cancer	Retrospective cohort study	428,478 newly diagnosed female patients with type II diabetes mellitus with onset at the age of 25 or later	Chemoprevention with sitagliptin	For cumulative duration and cumulative dose of sitagliptin use, the hazard ratios for the first and second tertiles were not significant, but a significantly reduced risk was observed in the third tertile of cumulative duration and cumulative dose. In the matched cohort, although the overall HR was not significant, a lower risk of breast cancer associated with sitagliptin use could also be observed. In the tertile analyses in the matched cohort, significantly reduced risk could also be observed for the third tertile of cumulative duration and cumulative dose.
97	[87]	Rosiglitazone reduces breast cancer risk in Taiwanese female patients with type 2 diabetes mellitus	2017	Taiwan	To study the association between rosiglitazone and breast cancer in Taiwanese female patients with type 2 diabetes mellitus	Retrospective cohort study	431,447 female patients with type II diabetes mellitus	Chemoprevention with oral antidiabetic agents or insulin	The overall hazard ratios for ever users versus never users showed a significantly lower risk in all models. While evaluating the dose–response relationship, a lower risk was observed in the third tertiles of both cumulative duration and cumulative dose in all models, and all *p* values for the trend were significant. There was a significant interaction between rosiglitazone and metformin. The lowest risk was observed in users of both drugs (hazard ratio 0.812, 95% confidence interval: 0.705–0.934).
110	[88]	Cost-effectiveness of surveillance and prevention strategies in BRCA1/2 mutation carriers	2018	Japan	This study aimed to conduct a cost-effectiveness analysis of surveillance and prevention strategies in BRCA1/2 mutation carriers with quality adjustment.	Economic evaluation	A simulated cohort of female BRCA1/2 mutation carriers aged 35–70 years, who had no cancer diagnosis at baseline	Risk-reducing mastectomy and risk-reducing salpingo-oophorectomy	Compared with surveillance, RRSO and RRM&RRSO were dominant (cost-saving and more effective) and RRM was also more cost-effective in BRCA1 mutation carriers. RRM and RRM&RRSO were dominant and RRSO was also more cost-effective in BRCA2 mutation carriers, with quality adjustment based on preference ratings.
111	[89]	Low-Dose Aspirin Reduces Breast Cancer Risk in Women with Diabetes: A Nationwide Retrospective Cohort Study in Taiwan	2017	Taiwan	This study aimed to investigate the relationship between low-dose aspirin use and breast cancer incidence in women with diabetes.	Retrospective cohort study	48,202 female patients with type II diabetes mellitus	Chemoprevention with aspirin	The risk of breast cancer was first found to be lower in aspirin users (HR, 0.77; 95% CI, 0.68–0.89; *p* < 0.01) in the unadjusted model. After adjustment for age, comorbidities, hypertension, and hyperlipidemia (Model 1), and modification of Model 1 with the addition of antidiabetic agents (Model 2), the risk of breast cancer was still found to be lower in aspirin users (HR, 0.78 and 0.81; *p* < 0.01 for Model 1 and Model 2, respectively). Only a high cumulative dose of aspirin (>88,900 mg) for a mean period of 8.5 years was found to reduce the risk of breast cancer (HR, 0.53; 95% CI, 0.43–0.67; *p* < 0.01). The incidence of breast cancer seemed to decrease after 1 year of aspirin use, with a continued divergence in the cumulative curves throughout the follow-up period.
112	[90]	Aspirin and Risk of Specific Breast Cancer Subtype in Women with Diabetes	2023	Taiwan	This study aimed to explore the association between aspirin use and the risk of specific breast cancer subtypes in women with diabetes.	Retrospective cohort study	48,202 female patients with type II diabetes mellitus	Chemoprevention with aspirin	The effect of ever use of aspirin appeared limited to hormone receptor-positive breast cancer. For ever use of aspirin, hormone receptor-positive breast cancer risk was significant (HR: 0.73; 95% CI: 0.59–0.91); however, it was not significant for hormone receptor-negative breast cancer risk (HR: 0.88; 95% CI: 0.74–1.05). A cumulative dose of aspirin use of more than 8600 mg for a mean period of 8.5 years was found to reduce the risk of hormone receptor-positive breast cancer by 31% (HR: 0.69; 95% CI: 0.50–0.97). A cumulative dose of aspirin use of more than 88,900 mg was found to reduce the risk of both hormone receptor-positive and hormone receptor-negative breast cancer. Among women aged ≤ 50 years old, aspirin use was associated with an increased risk of hormone receptor-positive breast cancer (HR: 1.44; 95% CI: 1.04–1.99). For women aged >50 years old, aspirin use was associated with decreased risk for both hormone receptor-positive and -negative breast cancer.

## Data Availability

No dataset was used for this scoping review. All information used from published sources is listed in Table 1.

## Supplementary Materials

The following supporting information can be downloaded at: https://www.mdpi.com/article/10.3390/cancers17020168/s1, Table S1: Search terms for Pubmed. Table S2: Search terms for Web of Science.
