# Supplementary material for: A Scoping Review of Primary Breast Cancer Risk Reduction Strategies in East and Southeast Asia
Source: Cancers (Basel). 2025 Jan 7;17(2):168. doi: 10.3390/cancers17020168 (PMC11763974; doi:10.3390/cancers17020168)
Supplement: Supplementary file 1 [file cancers-17-00168-s001.zip › cancers-3357820-supplementary.pdf]

Table S1. Search terms for Pubmed. An asterisk is a truncation symbol that helps find all possible endings of a word, allowing for broader search results (e.g., "breast" would include "breasts," "breast cancer," "breast malignancy"). The [tw] indicates that the search is restricted to the title and abstract (text words) fields of articles. The search strategy was developed iteratively, gradually incorporating important key terms and filters, and assessing how effectively they streamlined the search process.

Query	Search terms or keywords used	Results
#1	breast*[tw] OR breast cancer*[tw] OR breast neoplasm*[tw] OR breast malignancy*[tw] OR breast carcinoma*[tw]	646,186
#2	female*[tw] OR woman*[tw] OR women*[tw]	10,093,073
#3	risk*[tw] OR high risk*[tw] OR increased risk*[tw] OR heightened risk*[tw] OR above average risk*[tw]	3,589,652
#4	Asia*[tw] OR Southeast*[tw] OR South east*[tw] OR Brunei*[tw] OR Cambodia*[tw] OR China*[tw] OR Chine*[tw] OR Indonesia*[tw] OR Japan*[tw] OR Lao[tw] OR Malay*[tw] OR Mongoli*[tw] OR Myanmar*[tw] OR Burm*[tw] OR North Korea*[tw] OR Korea*[tw] OR Philippin*[tw] OR Filipin*[tw] OR Singapore*[tw] OR South Korea*[tw] OR Taiwan*[tw] OR Thai*[tw] OR Tibet*[tw] OR Vietnam*[tw]	1,528,154
#5	intervention*[tw] OR strateg*[tw] OR initiative*[tw] OR method*[tw] OR approach*[tw] OR guid*[tw] OR surger*[tw] OR mastectom*[tw] OR lifestyle*[tw] OR pharmac*[tw] OR Tamoxifen[tw] OR Raloxifene[tw] OR Aromatase[tw] OR chemop*[tw]	18,822,021
#6	prevention*[tw] OR risk reduc*[tw] OR primary prevention*[tw] OR lower*[tw] OR risk lower*[tw] OR prevent*[tw] OR control*[tw]	9,392,229
#7	#1 AND #4	30,171
#8	#1 AND #4 AND #3	9,834
#9	#1 AND #4 AND #3 AND #6	5,831
#10	#1 AND #4 AND #3 AND #6 AND #5 AND #2 (all queries)	3,609
#11	#1 AND #4 AND #3 AND #6 AND #5 AND #2 (all queries) AND Filters: English, Full-text	3,241
#12	#1 AND #4 AND #3 AND #6 AND #5 AND #2 (all queries) AND Filters: English, Full-text, from 2010/1-2024/07/01, Sort by: Most Recent	2,624

Table S2. Search terms for Web of Science. An asterisk is a truncation symbol that helps find all possible endings of a word, allowing for broader search results (e.g., "breast" would include "breasts," "breast cancer," "breast malignancy"). TS indicates that the search is restricted to topic terms, including the title, abstract, author keywords, and Keywords Plus®. Keywords Plus® consists of words or phrases that often appear in the titles of the references of a study, but do not appear in the article itself. The search strategy was developed iteratively, gradually incorporating important key terms and filters, and assessing how effectively they streamlined the search process.

Query	Search terms or keywords used	Results
#1	TS=(("breast*" OR "breast cancer*" OR "breast neoplasm*" OR "breast malignancy*" OR "breast carcinoma*"))	880,653
#2	TS=(("female*" OR "woman*" OR "women*"))	2,975,987

#3	TS=(("risk*" OR "high risk*" OR "increased risk*" OR "heightened risk*" OR "above average risk*"))	4,339,432
#4	TS=(("Asia*" OR "Southeast*" OR "South east*" OR "Brunei*" OR "Cambodia*" OR "China*" OR "Chine*" OR "Indonesia*" OR "Japan*" OR "Lao*" OR "Malay*" OR "Mongoli*" OR "Myanmar*" OR "Burm*" OR "North Korea*" OR "Korea*" OR "Philippin*" OR "Filipin*" OR "Singapore*" OR "South Korea*" OR "Taiwan*" OR "Thai*" OR "Tibet*" OR "Vietnam*"))	12,242,148
#5	TS=(("intervention*" OR "strateg*" OR "initiative*" OR "method*" OR "approach*" OR "guid*" OR "surger*" OR "mastectom*" OR "lifestyle*" OR "pharmac*" OR "Tamoxifen" OR "Raloxifene" OR "Aromatase" OR "chemop*"))	3,086,549
#6	TS=(("prevention*" OR "risk reduc*" OR "primary prevention*" OR "lower*" OR "risk lower*" OR "prevent*" OR "control*"))	21,555,799
#7	#1 AND #2 AND #3 AND #4 AND #5 AND #6, English, 2010 onwards	2,459
